# Dementia screening in Brazil: a systematic review of normative data for the mini-mental state examination

**DOI:** 10.6061/clinics/2019/e971

**Published:** 2019-10-30

**Authors:** Gustavo Santiago-Bravo, Felipe Kenji Sudo, Naima Assunção, Claudia Drummond, Paulo Mattos

**Affiliations:** I Memory Clinic, Instituto D’Or de Pesquisa e Ensino, Rio de Janeiro, RJ, BR; II Departamento de Psicologia, Pontificia Universidade Catolica do Rio de Janeiro, Rio de Janeiro, RJ, BR; III Programa de Pos Graduacao em Ciencias Morfologicas, Instituto de Ciencias Biologicas, Universidade Federal do Rio de Janeiro, Rio de Janeiro, RJ, BR; IV Departamento de Fonoaudiologia, Universidade Federal do Rio de Janeiro, Rio de Janeiro, RJ, BR; V Departamento de Psiquiatria e Medicina Forense, Instituto de Psiquiatria, Universidade Federal do Rio de Janeiro, Rio de Janeiro, RJ, BR

**Keywords:** Mental Status and Dementia Tests, Cognitive Dysfunction, Neurocognitive Disorders, Validation Studies

## Abstract

The Mini-Mental State Examination (MMSE) is the most widely used instrument for the screening of older adults with suspected cognitive impairment; the MMSE has been translated and validated in numerous languages and countries. The cultural and sociodemographic characteristics of the sample can influence performance on the test; therefore, applying the MMSE in different populations usually requires adjustments of those variables. From this perspective, the present study aims to review the normative data for the MMSE in the Brazilian older population, including those adaptations to the original test.

Database searches were performed in Medline, Web of Knowledge, Scielo and Pepsic for articles assessing healthy elderly Brazilian samples using the MMSE. Heterogeneity across and within the studies was analyzed.

Of a total of 1,085 retrieved articles, 14 were included. Significant differences across studies were identified for the characteristics of the samples, the presence of alterations to the MMSE subtests and the presentation of the results. The risk of biases was relevant for all the studies.

Considering the large methodological heterogeneity among studies, the generalization of the available normative data for the MMSE may not be appropriate for the general elderly Brazilian population.

## INTRODUCTION

Different levels of cognitive impairment are common among subjects 60 years old and over. The possible causes are heterogeneous, and there are often overlapping causes. According to recent epidemiological reports, 4.7 to 8.7% of elderly individuals may present with dementia, and as many as 42% may be diagnosed with mild cognitive impairment (MCI) (1,2). Although the benefits of routine testing for cognitive impairment have not been endorsed by international work groups, screening tests are recommended for the detection of cognitive impairment in those suspected of having Alzheimer’s disease (AD) or other conditions affecting cognition (3,4). In fact, the timely diagnosis of those disorders has been a major concern from public health authorities due to their exponential increase in prevalence over the last decades. The high socioeconomic cost and the deleterious impact on the quality of life of both patients and caregivers have also been focuses of consideration (1,5).

The rationale for the early detection of dementia (or MCI) is to allow clinical intervention in its initial stages and to anticipate medical, financial and legal decision making prior to the onset of mental incapacity (6). Regarding the first goal, however, the UK National Screening Committee published, in 2015, a statement positioned against the systematic assessment of elderly subjects, arguing that (i) cognitive screening tests are not sufficiently accurate for detecting subtle cognitive changes; (ii) outcomes for MCI can vary greatly, and more studies are needed to determine those who actually are at-risk for progressing to dementia; and (iii) there is no evidence for the advantages of early intervention on the natural history of MCI or dementia (7). Both the National Institute of Health and Care Excellence and the U.S. Preventive Services Task Force upheld similar recommendations (3,8); thus, primary care providers and geriatricians were uncertain whether the early detection of cognitive impairments in older patients with formal instruments was a feasible and valid practice.

The Mini-Mental State Examination (MMSE) is probably the most well studied screening test for cognitive impairments available. Folstein’s paper in which the tool was first introduced accounts for almost 50,000 citations in the Scopus database since its publication in 1975 (9). Such an impact could be attributed to its short administration time and ease of application. In addition, a recent meta-analysis indicated that the instrument presented a sensitivity of 85% and a specificity of 90% for the detection of dementia in community and primary care settings (10). On the other hand, accuracy for detecting dementia is significantly influenced by the stage of the disorder and the patient’s age, ethnic group and education (11). Adjusting cutoff values in accordance with those variables has resulted in different normative data for groups with specific sociodemographic features, including region or country of residence (12), severity of cognitive and functional symptoms (13) and educational level (14). Cross-cultural adaptations have also been adopted to minimize cultural biases (14).

In Brazil, several normative studies for the MMSE have been proposed throughout the years to distinguish normal aging from dementia. The present review aims to address Brazilian normative data for dementia in elderly samples and to evaluate the psychometric properties, cross-cultural differences and other methodological aspects of the MMSE that lead to divergences in the interpretation of results.

## METHODS

### Search strategy and selection of studies

In May 2018, database searches were conducted in Medline, Web of Knowledge, Scielo and Pepsic, using the terms MMSE OR Mini-Mental State Examination AND Brazil OR Brazilian. No limits were placed on the date of publication or field.

We included articles that provided normative data for the MMSE in subjects aged 60 years or over. Normative values were presented as the means and standard deviations, percentiles or quartiles. We excluded studies that (i) did not assess MMSE performance in a healthy elderly Brazilian sample; (ii) did not present differential scores according to age, sex, educational level, income or ethnicity; (iii) used modified versions of the MMSE (e.g., the Severe MMSE, Modified MMSE, MMSE-2^nd^ edition); (iv) assessed MMSE performance exclusively in subjects with cognitive impairment or dementia; and (v) consisted of posters, reviews, case reports or comments.

### Procedures

The screening of the retrieved articles and data extraction were independently performed by two of the authors (G.B. and F.K.S.). Divergences were discussed with the entire team of investigators, and decisions to include or exclude the articles were made by consensus. Quality assessment of the selected studies was appraised through a modified version of the QUADAS-2 by the same investigators (15).

## RESULTS

**Figure 1:**
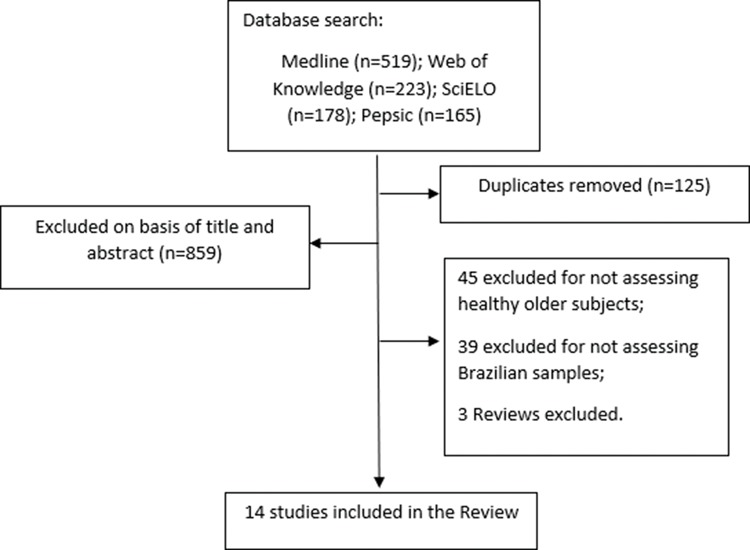
Flow diagram of study selection.

Of a total of 1,085 retrieved articles, 14 were selected for the present review. Figure 1 describes the stages for the selection of the studies.

### a) Recruitment

Fourteen studies presented normative data for the MMSE in older Brazilian samples. A total of 10,364 healthy older subjects were assessed in those studies, 74.3% of which were participants in 5 community-based studies (16-20), and 1 study was conducted with a mixed tertiary-community sample (21). The remaining subjects were convenience samples drawn from users of tertiary facilities (14,22-28). Six studies were conducted in the State of São Paulo (14,19,21,25,27,29), five were performed in Rio de Janeiro (16,17,19,20,23), one was placed in Minas Gerais (30), one was conducted in Pernambuco (26), and one was performed in Rio Grande do Sul (24). Studies assessed populations from the following cities: Catanduva/SP (21), Santo Antônio de Pádua/RJ (16,17), Bambuí/MG (18), São Paulo/SP (19,20), Ribeirão Preto/SP (19), Belém/PA (20), Parnaíba/PI (20), Campina Grande/PB (20), Poços de Caldas/MG (20), Campinas/SP (20) and Ivoti/RS (20).

### b) Age

Samples comprised elderly subjects (≥60 years old), with the exception of three studies, which also included younger subjects—middle-aged adults and/or adolescents (14,21,25). Bertolucci et al. (14) analyzed a mixed sample comprising both outpatients and hospitalized individuals, among which only 25% were older subjects. Among those studies presenting age groups, only one investigated the oldest-old persons, i.e., those aged 85 years or more (16). Although other studies included individuals aged 75 years or more, there were no data indicating how many of the oldest-old persons were included in those samples (19,20).

### c) Schooling

Schooling was depicted as a major factor affecting cognitive performance in all studies, and the mean scores for the test were stratified by years of formal education. The number of schooling-level clusters varied across studies, from as low as two (comparing illiterate to literate) (16,22,23) to as high as five groups of educational levels (21).

A threshold of eight years, equivalent to primary schooling, was the highest analyzed educational cluster in three of the studies (14,18,25), and the number of subjects with higher educational levels was not acknowledged in those papers.

### d) Diagnosis

With respect to the diagnostic groups assessed in the studies, Bertolucci et al. (14) compared normal controls, subjects with cognitive impairments of different etiologies (AD, normal pressure hydrocephalus, isolated memory deficits and unspecified cognitive impairments) and patients in a confusional state (according to the DSM-III-R) (14). In Almeida et al. (22), the sample comprised subjects with the following ICD-10 diagnoses: depressive disorders (40.3%); dementia (33.2%); neurotic, stress-related and somatoform disorders (9.5%); organic syndromes (4.7%); bipolar disorder (2.8%); dysthymia (2.8%); schizophrenia and delusional disorders (2.4%); substance use disorders (1.9%); and other diagnoses (2.4%). Brucki et al. (21) evaluated healthy individuals with no memory complaints (as determined by a structured questionnaire) who did not present signs and symptoms of neurological or psychiatric disorders. Subjects were classified as independent older individuals through a score lower than 5 on the Pfeffer’s Functional Activities Questionnaire in Laks et al. (17). Lourenço et al. (23) excluded subjects who could not understand and complete the test due to mental disorders or severe cognitive impairment, although individuals with depression (18.8% of the sample), Parkinson’s disease (1.3%) and dementia (25.74%) (DSM-IV and ICD-10) were included (23). Subjects with cognitive, sensorial or motor impairments were excluded from the study conducted by Sposito et al. (20). Women aged 60-79 years, whose scores on the MMSE were within the normal range for age and schooling according to cutoffs suggested by Brucki et al. (21), were included in the study by Cassimiro et al. (27). Other exclusion criteria in this study were the presence of functional difficulties (Functional Activities Questionnaire (FAQ) >1), neurologic deficits, history of alcohol or drug abuse, use of benzodiazepines, hypnotics or anticonvulsants and history of pathological gambling (27).

### e) Cross-cultural and other methodological adaptations

Although cross-cultural adaptations to the original instrument were not acknowledged in most of the studies (16,17,20,24,25,27,28), some changes on the MMSE subtests could be identified in others, as described below.

#### Orientation

Brucki et al. (21) proposed using “approximate time of the day” instead of “season”, “type of place” (hospital room, for example) instead of “floor” and “a nearby street” instead of “district”. The absence of marked environmental changes according to the season, as observed in tropical countries, was considered to impose a greater challenge for this task in Brazilian samples than in other populations. “Type of place” was suggested as an appropriate replacement for “floor”, considering that the instrument could be applied in different settings (community or inpatient services, for instance) (21). Likewise, Bertolucci et al. (14) chose “semester” instead of “season”. Although it was not mentioned, Castro-Costa et al. (18) may have changed “season” to “part of the day” and “floor” to “address” for similar reasons (18). Brito-Marques et al. (26) introduced the command in the orientation subtest: “ask the name of this place and point your finger down simultaneously” (26).

#### Attention

Brucki et al. (21) dropped the backward spelling of the word “mundo” since spelling is not as common in Brazilian culture as it is for American or Canadian populations (21). In contrast, Castro-Costa et al. (18) applied a backward spelling of “Maria” task, considering that this change would correspond to an equivalent level of difficulty to the “world” test for native English speakers. Those authors adopted serial additions of 5 beginning from zero instead of repeated subtractions of 7 from 100 to avoid floor effects among subjects with lower educational levels (18). Brito-Marques et al. (26) added a counting backward task from 25 as an alternative for the calculation task.

#### Memory

The words used in the registration/recall test were different across studies. The original English version does not specify the three objects to be memorized (9); hence, variations were observed among the articles. In general, authors chose nouns related to common concrete objects. For example, Brucki (21) used the terms “carro, vaso, tijolo”. The others adopted the words “tapete, tijolo, caneca”, except for Lourenço et al. (23), who chose the expressions “carro, vaso, bola”.

#### Language

Lourenço et al. (23) used a pencil for the naming task, while Brucki et al. (21) and Bertolucci et al. (14) recommended a pen. Some authors indicated that cues for the writing task could be provided to subjects; Brucki et al. (21) suggested writing “a sentence with beginning, middle and ending” or “something that has happened today”/“something that you want to say”, whereas Bertolucci et al. (14) would encourage patients to “write about the weather”. Notably, the original instruction for scoring this subtest recommended that the sentence should include a noun and a verb to be considered correct (9). The 3-stage command task (“take the paper in your right hand, fold it in half and put it on the floor”) also differed slightly in some studies: for the final command, Lourenço et al. (23) asked the participants to “throw it on the floor”, whereas Brito-Marques et al. (26) instructed the patients to give the paper to the investigator. Interestingly, the instructions for the repetition task (“repeat the phrase: no ifs, ands or buts”) were consistent across studies (repetition of “nem aqui, nem ali, nem lá”). Considering that the original sentence refers to a well-known expression among native English speakers, which is not the case for the Brazilian version, it is possible that the memory processes necessary to complete the Brazilian interpretation differ from the original task.

#### Praxis

The task of copying pentagons was changed to copying intersecting equilateral triangles by Brito-Marques et al. (26).

### f) Additional clustering of samples and presentation of the results

Cutoff values have varied largely across studies, which could be attributed to different sample profiles and predetermined stratification. Only 2 out of the 14 studies considered both schooling and age for stratification (16,17). One study considered schooling, age and sex, but analyses were independently performed, and relationships across the different categories were not explored (20).

MMSE performance stratified by age groups was found in five of the selected articles (16-20). One of the studies also stratified the sample by sex and income (20), whereas another study included data from only female subjects (27). While Bertolucci (14) used DSM-III-R criteria, all other used DSM-IV criteria for a dementia diagnosis. For illiterate individuals, the MMSE cutoff scores varied among the different studies and ranged from as low as 13 (14) to as high as 22 points (24).

The results were displayed as the means and standard deviations in most of the studies (16,19-21,25,26), while some studies employed quartiles and/or percentiles (17,18,27). Four of the studies did not acknowledge the standard deviations for the mean scores (14,22-24). The sensitivity and specificity of the MMSE were calculated in four of the studies (14,22-24).

Table 1 summarizes the characteristics and the results from the selected articles.

### g) Risk of biases

The representativeness of the sample may be questionable for most of the studies due to the wide age range (14,21,25), the inclusion of different diagnostic groups (17,22) and the use of convenience samples (14,22-28). Furthermore, potential biases associated with the application of the MMSE were identified in some studies. For instance, Castro-Costa et al. (18) adopted a significantly modified version of the MMSE; Moraes et al. (19) calculated the MMSE scores through the collection of the corresponding items within the Cambridge Mental Disorders of the Elderly Examination (CAMDEX); and Cassimiro et al. (27) and Leite et al. (28) adopted a prespecified cutoff value on the MMSE for the selection of the sample. Moreover, studies that assessed the accuracy of the instrument for detecting cognitive impairment may have suffered from the lack of a clinically validated standard reference for this condition (14,22-24). The lack of information on cross-cultural alterations to the MMSE made the analysis of the risk of bias associated with this issue unclear for most of the studies (16,17,20,24,25,27,28). As a result, none of the selected articles achieved high-quality status, according to the QUADAS-2 (15). The evaluation of the risk of bias within studies is depicted in Supplementary Table 1.

## DISCUSSION

The available evidence on normative values for the MMSE in the Brazilian elderly population shows high levels of heterogeneity concerning not only the methodological diversity among studies but also the array of modifications applied to the instrument by different authors. For this reason, the outcomes of the selected studies were not sufficiently comparable to perform a quantitative analysis of the thresholds and establish the accuracy of the MMSE for the evaluation of dementia in the Brazilian older population. Interstudy inconsistencies were detected for a variety of parameters, such as the selection of participants (exclusion/inclusion of young adults, for example) (14,21,25), the choice of factors included in the data analysis (effects of age, schooling or both on cognitive performance) (16-20), the presentation of the results (mean scores and standard deviations, accuracy calculations of proposed cutoffs or percentiles/quartiles distributions) (14,16,19-26) and the clustering criteria of the sample (as simple as comparing illiterate vs. literate groups to varying levels of education) (16,22,23). The validity of the results may have also been hampered by the divergent versions of the MMSE. These adaptations were used by researchers for multiple reasons, such as overcoming regional and cross-cultural differences between international samples and the Brazilian elderly population (e.g., replacement of “season” with “semester” or “part of the day”; removing the backward “world” task due to a lack of familiarity of Brazilians to spelling tasks) and to avoid floor effects related to educational bias (serial additions of “5” instead of subtractions of “7” and spelling “Maria” backward instead of “world”) (14,18,21,23,26).

In addition, the significant risk of bias within the individual studies should be discussed. Samples were not representative of the general Brazilian older population in most of the cases, which could be attributed to the recruitment of participants in specialized tertiary settings (14,22-28) or to the inclusion of young adults among the older subjects in some studies (14,21,25). The lack of calculations for determining sample sizes in the articles may raise doubts about the subsequent precision of the size effects estimated (29). Similarly, the generalization of outcomes could be disputable for the cluster arrangements, resulting in small numbers of participants, as depicted in some of the papers (17,21). Moreover, the analysis of the MMSE scores may have not been appropriately adjusted for potential confounders, such as the different diagnoses among the participants—presence of subjects with delirium (14) or other mental disorders (22)—and for the large ranges of ages within the groups (14,21). Finally, the use of nonvalidated modifications to the test, as previously described, may have also limited the reliability of the results (18,26).

A lack of harmonization of the screening methods for the initial detection of cognitive impairment may have been associated with delayed or missed diagnoses of morbid conditions. International studies have reported that up to 81% of subjects with dementia do not receive a diagnosis and are consequently deprived of the adequate treatment (30). To address this shortcoming, efforts to unify the concepts and procedures to identify dementia have been made by groups of experts. In Brazil, the Clinical Protocol and Therapeutic Guidelines for the diagnosis, the treatment and the follow-up of subjects with AD were published by the country’s Ministry of Health (MS) (31). According to this document, primary care physicians in public health facilities should adopt the MMSE and Clinical Dementia Rating (CDR) (32) as screening and staging instruments for subjects with suspected cognitive impairment (31). Patients with AD presenting MMSE scores between 8-21 (≤4 years of schooling) or 12-24 (>4 years of schooling) and CDR=1-2 (32) were eligible to receive cholinesterase inhibitors funded by the government. The diagnostic criteria for AD should be based on the 2011 guidelines published by the Brazilian Academy of Neurology (ABN), which endorsed the methods and normative scores for the MMSE (33,34) indicated by Brucki et al. (21) Strikingly, the cutoff scores illustrated in the MS protocol do not coincide with those presented in the suggested reference by the ABN (21), and the MS protocol thresholds have not undergone a validation process. Hence, although there is mounting evidence of the importance of early diagnosis of dementia, the recommended practices to formally assess this condition remain controversial (35).

To the best of our knowledge, this is the first study that systematically reviewed normative data from the MMSE for the detection of dementia in older Brazilian samples. Despite previous efforts to summarize the evidence regarding the use of this instrument in this population (36), the many distinctions concerning the scopes and approaches between the two studies are worth mentioning. Describing the translation and cross-cultural differences across MMSE versions might have disclosed confounding variables affecting results beyond the heterogeneity in educational levels among the samples. In addition, we have followed the recommendations portrayed in the PRISMA Statement for reporting systematic reviews (37); therefore, a broad search strategy in four different electronic databases was performed, allowing the retrieval of a comprehensive set of records in the field.

Investigations of the test’s psychometric properties are available for few of the individual adaptations. For instance, one study assessed the validity of one version of the MMSE against the clinicians’ impressions of the presence of dementia (23), whereas another study measured the test-retest reliability of the adapted version (38). Other researchers performed factor analyses, measuring correlations among clusters of MMSE subtests with those of international versions of the instrument (39,40). Therefore, discussing methodological inconsistencies across Brazilian MMSE variants may encourage greater attention for the need to redefine both clinical and research practices regarding the use of this tool in cognitive assessment**.**

Some limitations of the present review should be acknowledged. First, since studies have implied that the MMSE may show poor accuracy for the detection of dementia, especially in the early stages, we question whether the use of the MMSE should be further advocated, at least for research purposes (41). From this perspective and regarding the high cost of population-based studies, the relevance of new investigations focused on normative scores of the MMSE should be carefully judged by research ethics committees. Furthermore, establishing new parameters for the MMSE could be a misstep considering that the comparison analyses between the vast existing records and future data on test performance would be lost. One simple solution for this situation could be the validation of combined brief cognitive tasks, which include the MMSE with other tests, for use in the Brazilian elderly population (42). Establishing composite thresholds for the instruments may enhance the screening accuracy for cognitive impairment, while allowing individual consideration of the separate test scores.

Based on the currently available literature, we conclude that none of the normative scores for the MMSE could be recommended for the screening of subjects with dementia in Brazil. Issues both at the study level and related to the comparability across articles were identified in this review, leading to an inconsistent level of evidence for the use of any of the available data. Clinicians and researchers should be aware of these limitations when choosing to apply the MMSE for the assessment of older Brazilian subjects.

## APPENDIX

**Supplementary Table 1 -** Risk of biases within the studies

**Figure:**
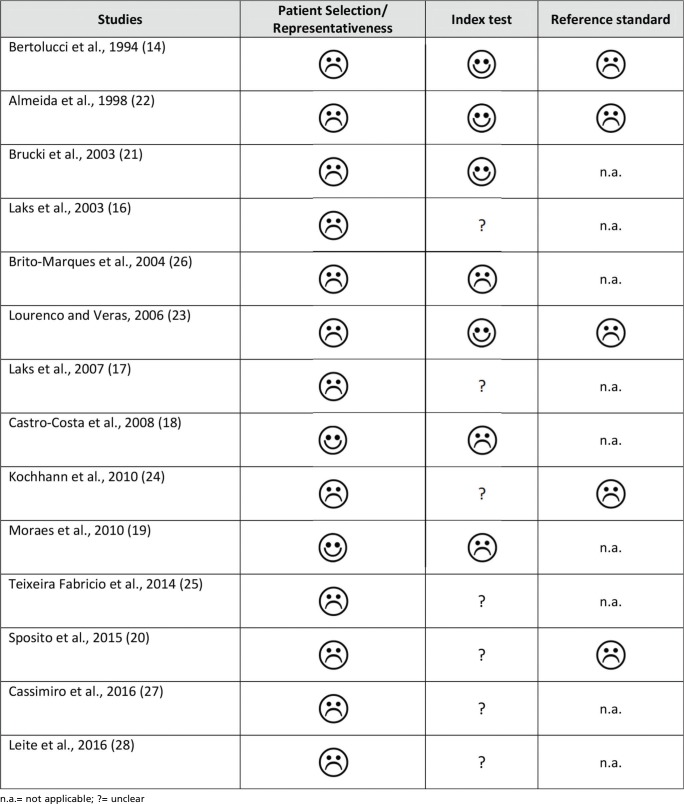


## AUTHOR CONTRIBUTIONS

Santiago-Bravo G, Sudo FK and Assunção N were responsible for the study conception, data curation, formal analysis, investigation, methodology procedures, resources and manuscript drafting. Drummond C and Mattos P are the senior authors and were responsible for the study conception, methodology procedures, project administration, resources, supervision, manuscript writing and review.

## Figures and Tables

**Table 1:** Summary of the selected studies.

Author, year	N	Age (years)	Setting, State	Groups	Cutoff	Sensitivity (%)	Specificity (%)
Bertolucci et al., 1994 (14)	530 healthy subjects, 94 in a confusional state (delirium or dementia)	51.75±15.5	Tertiary, SP	Illiterate	13	82.4	97.50
				1-7 years of schooling	18	75.6	96.6
				≥8 years of schooling	26	80.0	95.6
Almeida et al., 1998 (22)	211 healthy older subjects	69.38 (CI=68.32, 70.44)	Tertiary, SP	Illiterate	19	80.0	71.0
				Literate	23	77.8	75.4
Brucki et al., 2003 (21)	433 healthy subjects (convenience sample=289; community sample=144)	58.9±17.75	Community and Tertiary, SP	Illiterate	19.51±2.54 (20)		
				1-4 years of schooling	24.76±2.96 (25)		
				5-8 years of schooling	26.15±2.35 (26.5)		
				9-11 years of schooling	27.74±1.81 (28)		
				≥12 years of schooling	28.27±2.01 (29)		
Laks et al., 2003 (16)	341 healthy older subjects	74.01±6.31	Community, RJ	Illiterate: 65-84 years old	17.29±4.40 (13)		
				≥85 years old	14.33±3.89 (10.5)		
				Literate: 65-84 years old	22.42±4.98 (17.5)		
				≥85 years old	20.75±3.85		
Brito-Marques & Cabral-Filho, 2004 (26)	234 healthy older subjects	69.4±6.8	Tertiary, PE	Illiterate	18.86±4.59		
				1-4 years of schooling	22.98±3.47		
				5-8 years of schooling	23.98±3.39		
Lourenço et al., 2006 (23)	303 healthy older subjects	73.0±5.3	Tertiary, RJ	Illiterate	18	73.5	73.9
				Literate	24	75	69.7
Laks et al., 2007 (17)	397 healthy older subjects	72.15±7.24	Community, RJ	Illiterate: 65-74 years old	p5 = 14 (Q1 = 17)		
				75-84 years old	p5 = 13 (Q1 = 16)		
				1-8 years of schooling:65-74 years old	p5 = 15 (Q1 = 22)		
				75-84 years old	p5 = 15 (Q1 = 22)		
Castro-Costa et al., 2008 (18)	1,558 healthy older subjects	69.0±7.3	Community, MG	Illiterate: 60-64 years old	p5 = 13		
				≥65 years old	p5 = 10		
				1-7 years old years of schooling:60-64 years old	p5 = 20		
				≥ 65 years old	p5 = 16		
				≥ 8 years of schooling:60-64 years old	p5 = 25		
				≥ 65 years old	p5 = 24.5		
Kochhann et al., 2010 (24)	806 healthy older subjects and 162 subjects with dementia	70.6±7.3	Tertiary, RS	Illiterate	22	93	74
				1-5 years of schooling	22	87	82
				6-11 years of schooling	23	86	87
				≥ 12 years of schooling	24	81	87
Moraes et al., 2010 (19)	2,712 healthy older subjects	70.88±7.79	Community, RJ	Illiterate: < 75 years old	20.5±4.4		
				≥ 75 years old	17.2±5.5		
				1-4 years of schooling:< 75 years old	25.1±3.9		
				≥ 75 years old	22.9±5.3		
				≥ 5 years of schooling:< 75 years old	28.2±2.2		
				≥ 75 years old	26.2±4.4		
Teixeira Fabricio et al., 2014 (25)	180 healthy older subjects	64.98±6.79	Tertiary, SP	1-4 years of schooling	26.28±2.57		
				5-8 years of schooling	26.89±1.89		
				> 8 years of schooling	28.23±1.34		
Sposito et al., 2015 (20)	2,549 healthy older subjects	72.32±5.55	Community, RJ	Sex: Males	25.39±0.97		
				Females	24.74±0.99		
				Age: 65-69 years old	25.42±0.95		
				70-74 years old	25.03±1.00		
				75-79 years old	24.53±1.06		
				≥ 80 years old	24.12±1.04		
				Schooling: Illiterate	21.07±0.91		
				1-4 years of schooling	23.48±0.91		
				5-8 years of schooling	26.53±0.77		
				≥ 9 years of schooling	27.90±0.58		
				Income: <1.0 minimum wage	23.48±1.08		
				1.1-3.0 minimum wage	24.02±1.04		
				3.1-5.0 minimum wage	25.73±0.97		
				5.1-10.0 minimum wage	26.39±0.81		
				>10.0 minimum wage	27.61±0.65		
Cassimiro et al., 2016 (27)	164 healthy older women	Range: 66.5-73	Tertiary, SP	Illiterate	p25 = 20		
				1-2 years of schooling	p25 = 22		
				3-4 years of schooling	p25 = 23		
Leite et al., 2016 (28)	180 healthy older subjects	74.9±7.1	Tertiary, SP	Illiterate	20.1±2.2		
				1-2 years of schooling	24.4±2.1		
				3-4 years of schooling	24.6±2.1		

n.a.= not acknowledged.
